# HIF-1α Overexpression in Ductal Carcinoma In Situ of the Breast in *BRCA1* and *BRCA2* Mutation Carriers

**DOI:** 10.1371/journal.pone.0056055

**Published:** 2013-02-08

**Authors:** Petra van der Groep, Paul J. van Diest, Yvonne H. C. M. Smolders, Margreet G. E. M. Ausems, Rob B. van der Luijt, Fred H. Menko, Joost Bart, Elisabeth G. E. de Vries, Elsken van der Wall

**Affiliations:** 1 Department of Pathology, University Medical Center Utrecht, Utrecht, The Netherlands; 2 Department of Internal Medicine, University Medical Center Utrecht, Utrecht, The Netherlands; 3 Department of Medical Genetics, University Medical Center Utrecht, Utrecht, The Netherlands; 4 Department of Clinical Genetics, VU University Medical Center, Amsterdam, The Netherlands; 5 Department of Pathology, University of Groningen, University Medical Center Groningen, Groningen, The Netherlands; 6 Department of Medical Oncology, University of Groningen, University Medical Center Groningen, Groningen, The Netherlands; Ohio State University Medical Center, United States of America

## Abstract

Recent studies have revealed that *BRCA1* and *BRCA2* germline mutation-related breast cancers show frequent overexpression of hypoxia inducible factor-1α (HIF-1α), the key regulator of the hypoxia response. However, the question remained whether hypoxia is a late stage bystander or a true carcinogenetic event in patients with hereditary predisposition. We therefore studied HIF-1α overexpression in ductal carcinoma *in situ* (DCIS), an established precursor of invasive breast cancer.

We used immunohistochemistry to examine the expression of the hypoxia markers HIF-1α, CAIX and Glut-1 in DCIS and available invasive carcinoma lesions of 32 *BRCA1*, 16 *BRCA2* and 77 non-*BRCA* mutation-related cases. HIF-1α expression was detected in 63% of *BRCA1* and 62% of *BRCA2* as compared to 34% of non-*BRCA* mutation-related DCIS cases (p = 0.005). CAIX overexpression was present in 56% of *BRCA1* and 44% of *BRCA2* as compared to 6% of non-*BRCA* mutation-related DCIS cases (p = 0.000). Glut-1 overexpression was observed in 59% of *BRCA1*, 75% of *BRCA2* and 67% of non-*BRCA* mutation-related DCIS cases (p = 0.527). Overall, HIF-1α, CAIX and Glut-1 expression in *BRCA* mutation-related DCIS matched the expression in the accompanying invasive cancers in 60% or more of cases. In non-*BRCA* mutation-related cases the expression of the hypoxia markers in DCIS matched the expression in the invasive part in 46% or more of the cases.

Although *BRCA1* and *BRCA2* germline mutation-related invasive breast cancers are different in many ways, the hypoxia-related proteins HIF-1α, CAIX and Glut-1 are expressed in both DCIS and invasive lesions of *BRCA1* and *BRCA2* mutation carriers. This suggests that hypoxia may already play a role in the DCIS stage of *BRCA1* and *BRCA2* germline mutation related breast carcinogenesis, and may also drive cancer progression. Hypoxia-related proteins are therefore putative targets for therapy and molecular imaging for early detection and monitoring therapy response in *BRCA* mutation patients.

## Introduction

Hereditary breast cancer accounts for about 5% of all breast cancers in women and is primarily caused by a germline mutation in one of the *BRCA* genes. Several studies have indicated that the genetic makeup of *BRCA1* and *BRCA2* mutation-related breast cancer is different from that of non-*BRCA* mutation-related breast cancer. These differences comprise gains and losses of specific parts of chromosomes, as well as differences in protein expression [1]–[7]. Consistent with this, the morphological and immunohistochemical phenotype of *BRCA1* mutation-related breast cancer is also different from that of non-*BRCA* mutation-related breast [8]–[13]. However, the phenotype of *BRCA2* mutation-related breast cancer is still difficult to distinguish from non-*BRCA* mutation-related breast cancers [14], [15].

Hypoxia is a hallmark of many non-*BRCA* mutation-related breast cancer types [16]. Hypoxia inducible factor-1 (HIF-1) is the key regulator of the hypoxia response. HIF-1 consists of 2 subunits, HIF-1α and HIF-1β. While HIF-1β is constitutively expressed, the HIF-1α protein is continuously degraded under normoxia by the ubiquitin-proteasome pathway [17], [18]. Under hypoxia, HIF-1α protein degradation is inhibited resulting in its overexpression, subsequent binding to HIF-1β [19] and downstream signalling [20]. In non-*BRCA* mutation-related breast cancer, HIF-1α overexpression plays a role in carcinogenesis [21]–[26] and correlates with poor prognosis [27], [28]. When HIF-1α is overexpressed, established downstream targets like Carbonic anhydrase IX (CAIX) and Glucose transporter-1 (Glut-1) are also up regulated [29], [30]. *BRCA1* seems to play a role in the hypoxic response by regulating HIF-1α stability and by modulating expression of vascular endothelial growth factor, a major downstream target of HIF-1α [31]. Furthermore, functional HIF-1α overexpression (mostly hypoxia induced) is seen at a much higher frequency in *BRCA1* mutation-related invasive breast cancer than in sporadic breast cancer [32], [33]. In contrast, *BRCA2* mutation-related invasive cancers express HIF-1α less frequently [33].

However, studies in pre-invasive lesions are required to address the question whether hypoxia is a late stage bystander or a true carcinogenetic event.

There is both clinical and experimental evidence to suggest that ductal carcinoma *in situ* (DCIS) is a precursor lesion to most, if not all, non-*BRCA* mutation-related invasive breast cancers [34]–[38]. DCIS and other premalignant lesions such as lobular neoplasia, fibroadenoma, and ductal hyperplasia seems to be more common in prophylactic mastectomy (PM) specimens of *BRCA1* and *BRCA2* mutation carriers than in control mammoplasty specimens [10], [39]–[42]. Furthermore, DCIS lesions adjacent to invasive cancers in *BRCA* mutation carriers have been described [43], [44]. DCIS in *BRCA* mutation carriers is often high grade [43] and shows a similar morphology and immunophenotype as the accompanying invasive cancer [45]. High grade DCIS of non-*BRCA-*related cases often shows central necrosis [46] indicative of hypoxia. Indeed, overexpression of hypoxia-related proteins HIF-1α, CAIX and Glut-1 DCIS of non-*BRCA* mutation carriers has been described [22]. To find clues whether changes in hypoxia related proteins also is an early event in *BRCA* mutation-related carcinogenesis, we evaluated HIF-1α expression in *BRCA1* and *BRCA2* mutation-related DCIS in relation with the accompanying invasive cancers.

## Materials and Methods

### Patients

The study group comprised DCIS lesions of 32 patients with pathogenic germline *BRCA1* mutations, 16 patients with pathogenic germline *BRCA2* mutations and 77 patients unselected for family history (further denoted “non-*BRCA* mutation-related”). A synchronous invasive tumor was also present in 28 *BRCA1*, 17 *BRCA2* and 50 non-*BRCA* mutation-related cases. Tissue from these patients was available from our own archives, and from different pathology laboratories in The Netherlands (St Antonius Hospital Nieuwegein, Diakonessenhuis Utrecht, Gelre Ziekenhuizen Apeldoorn, Rijnstate Arnhem, Stichting Pathologisch en Cytologisch laboratorium West Brabant Bergen op Zoom, Ziekenhuis Gelderse Vallei Ede, Deventer Ziekenhuis Deventer, Meander medisch centrum Amersfoort, Onze Lieve Vrouwe Gasthuis Amsterdam, the VU University Medical Center, Amsterdam and the University Medical Center Groningen). Since we used archival pathology material which does not interfere with patient care and does not involve the physical involvement of the patient, no ethical approval is required according to Dutch legislation [the Medical Research Involving Human Subjects Act (Wet medisch-wetenschappelijk onderzoek met mensen, WMO [47])]. Use of anonymous or coded left over material for scientific purposes is part of the standard treatment contract with patients and therefore informed consent procedure was not required according to our institutional medical ethical review board. This has also been described by van Diest et al. [48].

### Histopathology

Tumor size was measured in the fresh resection specimens, and tumor samples were subsequently fixed in neutral buffered formaldehyde, and processed to paraffin blocks according to standard procedures. Four µm thick sections were cut and stained with H&E for histopathology. Tumor type was assessed according to the WHO 2003, and tumors were graded according to the Nottingham grading system. Mitoses counting was performed as previously described [49]. Scoring was performed by one observer (PJvD) who was blinded to the origin of the tumors.

### Immunohistochemistry

After deparaffinization and rehydration, antigen retrieval was performed using EDTA buffer at boiling temperature for 20 minutes for ER, HER2 and HIF-1α. A cooling period of 30 minutes preceded the incubation of the slides for HIF-1α with protein block (Novolink Max Polymer detection system, ready to use, Novocastra Laboratories Ltd, Newcastle Upon Tyne, UK) for 5 minutes at room temperature. Incubation of the slides with the HIF-1α mouse monoclonal (BD Biosciences, Pharmingen, Lexington, MA, USA), was done at a dilution of 1∶50 overnight at 4°C. For detection, a polymer (Novolink Max Polymer detection system, ready to use) was used. For ER and HER2, the slides were incubated with primary antibodies for ER (1∶100, Dako) and HER2 (1∶100, Neomarkers) 60 minutes at room temperature.

For PR, Glut-1 and CAIX, antigen retrieval was performed in citrate buffer, pH = 6.0, for 20 minutes at 100°C. A cooling period of 30 minutes preceded the incubation (60 minutes at room temperature) with the primary antibodies. Polyclonal primary antibodies used were: PR (1∶100, Dako), Glut-1 (1∶200, DAKO) and CAIX (1∶1000, Abcam, Cambridge Science Park, Cambridge, UK). For detection of the primary antibodies against ER, PR, HER2, CAIX and Glut-1, a poly HRP anti- Mouse/Rabbit/Rat IgG (ready to use, ImmunoLogic, Duiven, Netherlands) was used. All slides were developed with diaminobenzidine (10 minutes) followed by hematoxylin counterstaining. Before the slides were mounted all sections were dehydrated in alcohol and xylene. Positive controls were used throughout, negative controls were obtained by omission of the primary antibodies from the staining procedure. Representative pictures of positive and negative controls for HIF-1α, CAIX and Glut-1 have been provided as Figure S1.

Scoring of immunohistochemistry was performed by one observer (PJvD). HIF-1α was regarded overexpressed when >1% of nuclei were positive as described before [26]. ER and PR expression was regarded positive when 10% or more of the tumor nuclei stained positive. HER2 was scored positive when a 3+ membrane staining was observed according to the Dako system. CAIX and Glut-1 stainings were scored positive when a clear membrane staining pattern was seen. Associations between stainings were tested by Chi-square analysis. P-values<0.05 were considered to be statistically significant.

## Results

The clinicopathological characteristics and expression of ER, PR, HER2, HIF-1α, CAIX and Glut-1 of *BRCA1*, *BRCA2 and non-BRCA* mutation-related DCIS cases are described in Table 1. The age of onset is lower in *BRCA* compared to non-*BRCA* mutation carriers (p = 0.000). *BRCA1* mutation-related DCIS cases often are ER, PR and HER2-negative as compared to the *BRCA2* and non-*BRCA* mutation-related DCIS (see Table 1 for correlations).

**Table 1:** Clinicopathological characteristics and expression of ER, PR, HER2, HIF-1α, CAIX and Glut-1 in DCIS lesions of *BRCA1*, *BRCA2* and non-*BRCA* mutation carriers.

		*BRCA1*	*BRCA2*	non-*BRCA*	p-value
	N	32	16	77	
Age	<45	25(78%)	9(56%)	14(18%)	
	>45	7(22%)	7(44%)	63(82%)	0.000
Grade	1	0(0%)	1(6%)	11(14%)	
	2	9(28%)	8(50%)	30(39%)	
	3	23(72%)	7(44%)	36(47%)	0.035
ER	neg	22(69%)	4(25%)	19(25%)	
	pos	10(31%)	12(75%)	58(75%)	0.000
PR	neg	27(84%)	9(56%)	36(47%)	
	pos	5(16%)	7(44%)	41(53%)	0.002
HER2	neg	31(97%)	11(69%)	55(71%)	
	pos	1(3%)	5(31%)	22(29%)	0.014
HIF-1α	neg	12(38%)	6(38%)	51(66%)	
	pos	20(63%)	10(62%)	26(34%)	0.005
CAIX	neg	14(44%)	9(56%)	72(94%)	
	pos	18(56%)	7(44%)	5(6%)	0.000
Glut-1	neg	13(41%)	4(25%)	25(33%)	
	pos	19(59%)	12(75%)	52(67%)	0.527

### Expression of hypoxia-induced proteins in BRCA1, BRCA2 and non-BRCA mutation-related DCIS

HIF-1α overexpression was observed in 63% (20/32) of the *BRCA1*, in 62% (10/16) of the *BRCA2* and in 34% (26/77) of the non-*BRCA* mutation-related DCIS cases (p = 0.005;Table 1).

CAIX overexpression was observed in 56% (18/32) of *BRCA1* mutation-related DCIS cases, with accompanying HIF-1α overexpression in 31% (10/32) of the cases (p = 0.358;Table 2). Glut-1 was overexpressed in 59% (19/32) of the *BRCA1* mutation-related DCIS cases and HIF-1α was co-overexpression in 41% (13/32) of these cases (p = 0.403).

**Table 2:** Correlation of HIF-1α expression in DCIS lesions of *BRCA1*, *BRCA2* and non-*BRCA* mutation carriers with age, grade, ER, PR, HER2, CAIX and Glut-1 expression in these lesions.

		*BRCA1*			*BRCA2*			non-*BRCA*		
	N	32			16			77		
		HIF-1α		p-value	HIF-1α		p-value	HIF-1α		p-value
		neg	pos		neg	pos		neg	pos	
Age	<45	9	16		3	6		8	6	
	>45	3	4	0.740	3	4	0.696	43	20	0.427
Grade	1	0	0		1	0		10	1	
	2	2	7		4	4		21	9	
	3	10	13	0.264	1	6	0.149	20	16	0.081
ER	neg	8	14		1	3		9	10	
	pos	4	6	0.844	5	7	0.551	42	16	0.045
PR	neg	10	17		4	5		22	14	
	pos	2	3	0.900	2	5	0.515	29	12	0.373
HER2	neg	12	19		5	6		39	16	
	pos	0	1	0.431	1	4	0.330	12	10	0.170
CAIX	neg	4	10		5	4		46	26	
	pos	8	10	0.358	1	6	0.091	5	0	0.099
Glut-1	neg	6	7		3	1		21	4	
	pos	6	13	0.403	3	9	0.074	30	22	0.022

CAIX was expressed in 44% (7/16) of *BRCA2* mutation-related DCIS cases with accompanying HIF-1α overexpression in 38% (6/16) of the cases (p = 0.091). Glut-1 overexpression was observed in 75% (12/16) of *BRCA2* mutation-related DCIS cases, with HIF-1α co-overexpression in 56% (9/16) of the cases (p = 0.074).

In the non-*BRCA* mutation-related DCIS cases, CAIX expression was seen in 6% (5/77) of the cases which were negative for HIF-1α. Glut-1 was overexpressed in 67% (52/77) of non-*BRCA* mutation-related DCIS cases, with concomitant HIF-1α overexpression in 29% (22/77) of the cases (p = 0.022).

Furthermore, in the *BRCA1* and *BRCA2* mutation-related DCIS no correlations between HIF-1α expression and grade, ER, PR and HER2 expression were found. For the non-*BRCA* mutation-related DCIS cases, a positive trend was observed with grade, and a negative trend with ER (Table 2).

### Expression of hypoxia-induced proteins in BRCA1, BRCA2 and non-BRCA mutation-related DCIS and invasive cancer

**Figure 1:**
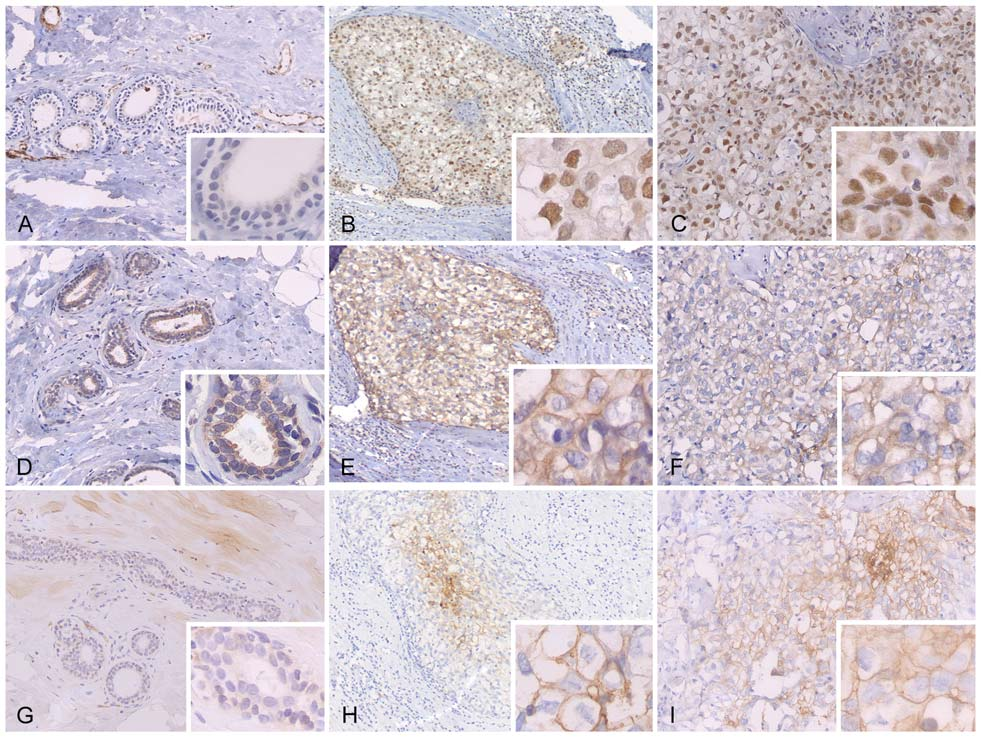
Immunohistochemical staining of HIF-1α, CAIX and Glut-1 in normal breast tissue (A, D and G), DCIS (B, E and H) and concomitant invasive cancer (C, F and I) of a *BRCA1* mutation carrier.

In the *BRCA1* mutation-related cases with DCIS and concomitant invasive cancer (N = 29), the frequency of HIF-1α overexpression was high in both lesions: 62% (18/29) and 83% (24/29), respectively (p = 0.264;Table 3.). The frequency of CAIX expression was 52% (15/29) and 79% (23/29), respectively, in DCIS and invasive carcinoma (p = 0.311). Further, 59% (17/29) of the DCIS and 83% (24/29) (p = 0.945) of the invasive lesions were positive for Glut-1 expression. Examples of these IHC results are shown in Figure 1.

**Table 3:** Clinicopathological characteristics and expression of ER, PR, HER2, HIF-1α, CAIX and Glut-1 in DCIS and accompanying invasive lesions of *BRCA1*, *BRCA2* and non-*BRCA* mutation carriers.

		Invasive			DCIS		
		*BRCA1*	*BRCA2*	non-*BRCA*	*BRCA1*	*BRCA2*	non-*BRCA*
	N	29	16	50	29	16	50
Age	<45	21	8	8			
	>45	8	8	42			
Type	ductal	27	15	45			
	lobular	1	0	3			
	other	1	1	2			
Grade	1	0	1	8	0	1	7
	2	4	7	20	8	8	19
	3	25	8	22	21	7	24
ER	neg	22	4	13	21	4	13
	pos	7	12	37	8	12	37
PR	neg	25	5	19	25	9	26
	pos	4	11	31	4	7	24
HER2	neg	26	13	39	28	11	33
	pos	3	3	11	1	5	17
HIF-1α	neg	5	10	33	11	6	31
	pos	24	6	17	18	10	19
CAIX	neg	6	9	44	14	9	46
	pos	23	7	6	15	7	4
Glut-1	neg	5	7	32	12	4	15
	pos	24	9	18	17	12	35

In the *BRCA2* mutation-related cases with invasive counterparts (N = 16), 63% (10/16) of DCIS lesions were HIF-1α positive as compared to 38% (6/16) if invasive lesions (p = 0.016). The same expression of CAIX was observed in *BRCA2* mutation-related DCIS lesions and the invasive counterpart lesions, 44% (7/16) (p = 0.049). Glut-1 was overexpressed in 75% (12/16) of DCIS cases in and in 56% (9/16) (p = 0.146) of the invasive *BRCA2* mutation-related lesions (Table 3).

The frequency of HIF-1α expression in non-*BRCA* mutation-related DCIS and concomitant invasive cancer (N = 50) was 38% (19/50) and 34% (17/50), respectively (p = 0.029). Similar CAIX expression was observed in both lesions, 8% (4/50) and 12% (6/50), respectively (p = 0.015). Glut-1 overexpression was seen in 70% (35/50) of DCIS cases and in 36% (18/50) (p = 0.797) of the invasive non-*BRCA* mutation-related lesions.

In summary, these non-significant differences indicate that HIF-1α positivity was similar in DCIS and the accompanying invasive lesions. Differences in HIF-1α expression between *BRCA1* and *BRCA2* and non-*BRCA* mutation related DCIS were borderline significant (p = 0.062). A significant difference in HIF-1α expression was seen between *BRCA1* and *BRCA2* as compared to non-*BRCA* mutation-related invasive cancer (p = 0.000).

### Expression of hypoxia-induced proteins in BRCA non-BRCA mutation-related DCIS vs invasive cancer

Table 4 shows the expression of HIF-1α, CAIX and Glut-1 in paired, DCIS and concomitant invasive cancer, for *BRCA* mutation and non-*BRCA* mutation carriers.

**Table 4:** Correlation of HIF-1α, CAIX and Glut-1 between invasive and DCIS lesions of *BRCA1*, *BRCA2* and non-*BRCA* mutation carriers.

*BRCA1*		Invasive								
		HIF-1alpha			CAIX			Glut-1		
		neg	pos	p-value	neg	pos	p-value	neg	pos	p-value
DCIS	neg	3	8		4	10		2	10	
	pos	2	16	0.264	2	13	0.311	3	14	0.945

*BRCA2*		Invasive								
		HIF-1alpha			CAIX			Glut-1		
		neg	pos	p-value	neg	pos	p-value	neg	pos	p-value
DCIS	neg	6	0		7	2		3	1	
	pos	4	6	0.016	2	5	0.049	4	8	0.146

non-*BRCA*		Invasive								
		HIF-1alpha			CAIX			Glut-1		
		neg	pos	p-value	neg	pos	p-value	neg	pos	p-value
DCIS	neg	24	7		42	4		10	5	
	pos	9	10	0.029	2	2	0.015	22	13	0.797

HIF-1α expression was expressed in both lesions in 55% (16/29) of the *BRCA1* mutation-related cases, whereas both lesions were negative for HIF-1α expression in 10% (3/29) of cases. Overall, in 66% (19/29) of the *BRCA1* mutation carrier cases both lesions showed similar expression levels of HIF-1α. In 28% (8/29) of the *BRCA1* mutation-related cases only the invasive part, and in 7% (2/29) only the DCIS lesion showed HIF-1α expression. CAIX and Glut1 were expressed in both lesions in 45% (13/29) and 48% (14/29) of the *BRCA1* mutation carrier cases, respectively, and both lesions lacked expression of these markers in 14% (4/29) and 7%(2/29) of the cases. Thereby, CAIX was concomitantly expressed in both lesions 59% (17/29) of the cases, and the Glut-1 in 55% (16/29). Only the invasive lesion of *BRCA1* mutation carriers expressed both CAIX and Glut-1 in 34% (10/29) of cases. Expression of CAIX and Glut-1 exclusively in *BRCA1* mutation-related DCIS lesions was observed in 7% (2/29) and 10% (3/29) of cases, respectively.

In the *BRCA2* mutation-related cases with DCIS and concomitant invasive cancer, 38% (6/16) of the cases HIF-1α expression was observed and was absent in 38% (6/16) of the cases (Table 4). Thus, in 75% (12/16) of the *BRCA2* mutation-related cases, the DCIS and invasive lesions of the same patient showed similar expression levels of HIF-1α. Expression of HIF-1α in only the DCIS lesion was seen in 25% (4/16) of the *BRCA2* mutation-related cases. CAIX was expressed in both lesions in 31% (5/16) of *BRCA2* mutation-related cases and in 44% (7/16) of the cases both lesions lacked expression (total match 75%). CAIX was expressed in the invasive, but not in the DCIS part in 13% (2/16) of the cases, and CAIX was expressed in the DCIS, but not in the invasive part of 13% (2/16) of the cases. Glut-1 was expressed or absent in both lesions in 50% (8/16) and 19% (3/16) of cases, respectively (total match 69%). Further, Glut-1 expression was confined to the invasive part in 6% (1/16) of cases and the DCIS part in 25% (4/16) of the cases.

HIF-1α was expressed in both lesions in 20% (10/50) of the non-*BRCA* mutation-related cases and both lesions lacked HIF-1α expression in 48% (24/50) of cases. Thus, in total, 68% (34/50) of the non-*BRCA* mutation carrier cases showed similar expression levels of HIF-1α in both lesions. In 14% (7/50) of the non-*BRCA* mutation-related cases only the invasive part, and in 18% (9/50) only the DCIS lesion showed HIF-1α expression. CAIX and Glut-1 were expressed in both lesions in 4% (2/50) and 26% (13/50), respectively, of the non-*BRCA* mutation carrier cases. Conversely, both lesions lacked CAIX expression in 84% (42/50) and Glut-1 expresssion in 20%(10/50) of these cases. Thereby, CAIX expression in both lesions matched in 88% (44/50) and Glut-1 expression in 46% (23/50) of cases. Expression of CAIX and Glut-1 in only the invasive lesion of non-*BRCA* mutation carriers occurred in 8% (4/50) and 10% (5/50) of cases, respectively, whereas these markers were expressed only in DCIS lesions in 4% (2/50) and 44% (22/50) of cases.

When *BRCA1* and *BRCA2* mutation-related cases were examined together, HIF-1α expression in DCIS matched the expression in the accompanying invasive cancers in 68% (31/45) of cases, as compared to in 68% (34/50) of the non-*BRCA* mutation carrier cases. The expression of CAIX matched in 64% (29/45) of *BRCA1* and *BRCA2* mutation-related cases, as compared to in 88% (44/50) of non-*BRCA* mutation carrier cases. For Glut-1, the expression in DCIS matched the expression in the accompanying invasive cancers in 60% (27/45) of *BRCA1* and *BRCA2* mutation-related cases as compared to 46% (23/50) for non-*BRCA* mutation carrier cases.

## Discussion

Non-*BRCA* mutation-related DCIS lesions, especially high grade ones, are known to become centrally deprived of oxygen resulting in activation of the hypoxia pathway, as shown in several studies by the presence of HIF-1α and its downstream targets. The aim of the present study was to examine the expression of HIF-1α in DCIS lesions of *BRCA1* and *BRCA2* mutation carriers in comparison with their invasive counterparts. Activation of HIF-1α in the DCIS stage of *BRCA1* or *BRCA2* germline mutated patients would indicate that hypoxia is an early driver of *BRCA* mutation-related carcinogenesis. HIF-1α overexpression was indeed frequently observed in *BRCA1* and *BRCA2* mutation-related DCIS cases, in association with expression of its downstream genes, indicating that HIF-1α is active.

Overall, 63% (30/48) of *BRCA* mutation-related DCIS lesions were HIF-1α-positive, which was significantly different compared to non-*BRCA* mutation carriers (34%, 26/77). The latter figure is lower compared to our earlier observations where 67% of sporadic DCIS lesions were HIF-1α positive [22]. Nevertheless, the current study suggests that hypoxia and HIF-1α already play a similar role in the DCIS stage of *BRCA* mutation-related carcinogenesis as in non-*BRCA* mutation-related DCIS.

*BRCA* mutation-related invasive cancers (especially *BRCA1* mutation-related ones) more frequently show HIF-1α overexpression than non-*BRCA* mutation-related ones [33], [34]. This suggests that hypoxia plays a more important role in cancer progression in *BRCA* mutation carriers than in non-*BRCA* mutation carriers. HIF-1α, CAIX and Glut-1 expression in *BRCA* mutation-related DCIS was usually similar in the accompanying invasive lesions. This implies that next to being involved in early *BRCA* mutation-related carcinogenesis, hypoxia and HIF-1α overexpression may also be a driver of cancer progression, especially in *BRCA1* mutation carriers. Although the number of *BRCA2* mutation-related cases with DCIS and invasive lesions was small, there was a trend towards higher expression of the hypoxia-related markers in *BRCA2* mutation-related DCIS as compared to the invasive lesions. We can speculate that progression to the invasive state in these *BRCA2* mutation carriers might be due to the switch of the HIF-1α to HIF-2α expression under prolonged hypoxia [50]. HIF-2α expression has been observed in sporadic breast cancer [51] and should be analysed in *BRCA* mutation-related breast cancer and pre-invasive lesions. As HIF-1α already plays a role in the pre-invasive lesions of *BRCA* mutation carriers, hypoxia proteins would therefore be putative therapeutic targets for prevention of invasive disease. HIF-1α signalling inhibitors like PX-478 [52], farnesyltransferase inhibitor R115777 or trans-farnesylthiosalicyclic acid [53], [54], Cetuximab [55] and other antibodies with the same structural motif [56], 2-methoxyestradiol (2ME2) [57], [58], and inhibitors of the RNA binding protein Hur [59], [60] are some of the therapeutics currently available.

We conclude that *BRCA1* and *BRCA2* germline mutation-related DCIS show a high frequency of overexpression of HIF-1α and its downstream proteins CAIX and Glut-1, as compared to non-*BRCA* mutation-related DCIS. This suggests that hypoxia may already play a role at the DCIS stage of *BRCA1* and *BRCA2* germline mutation-related breast carcinogenesis, and may also drive cancer progression. The current findings could be clinically relevant for *BRCA* mutation- related breast cancer treatment in several ways. First, HIF-1α and its downstream effectors may be used as molecular imaging targets for early detection and monitoring of therapy response. Second, HIF-1α is an interesting therapeutic target at the pre-invasive stage of *BRCA* mutation-related breast disease to prevent invasive disease.

## Supporting Information

Figure S1 Positive controls: Immunohistochemical staining of HIF-1α and CAIX in renal clear cell carcinoma (B and D) and for Glut-1 in placental tissue (F). In A, C and E the primary antibody was omitted to provide negative controls.
(TIF)
