# Supplementary figures and images for: HIF-1α Overexpression in Ductal Carcinoma In Situ of the Breast in BRCA1 and BRCA2 Mutation Carriers
Source: PLoS One. 2013 Feb 8;8(2):e56055. doi: 10.1371/journal.pone.0056055 (PMC3568038; doi:10.1371/journal.pone.0056055)

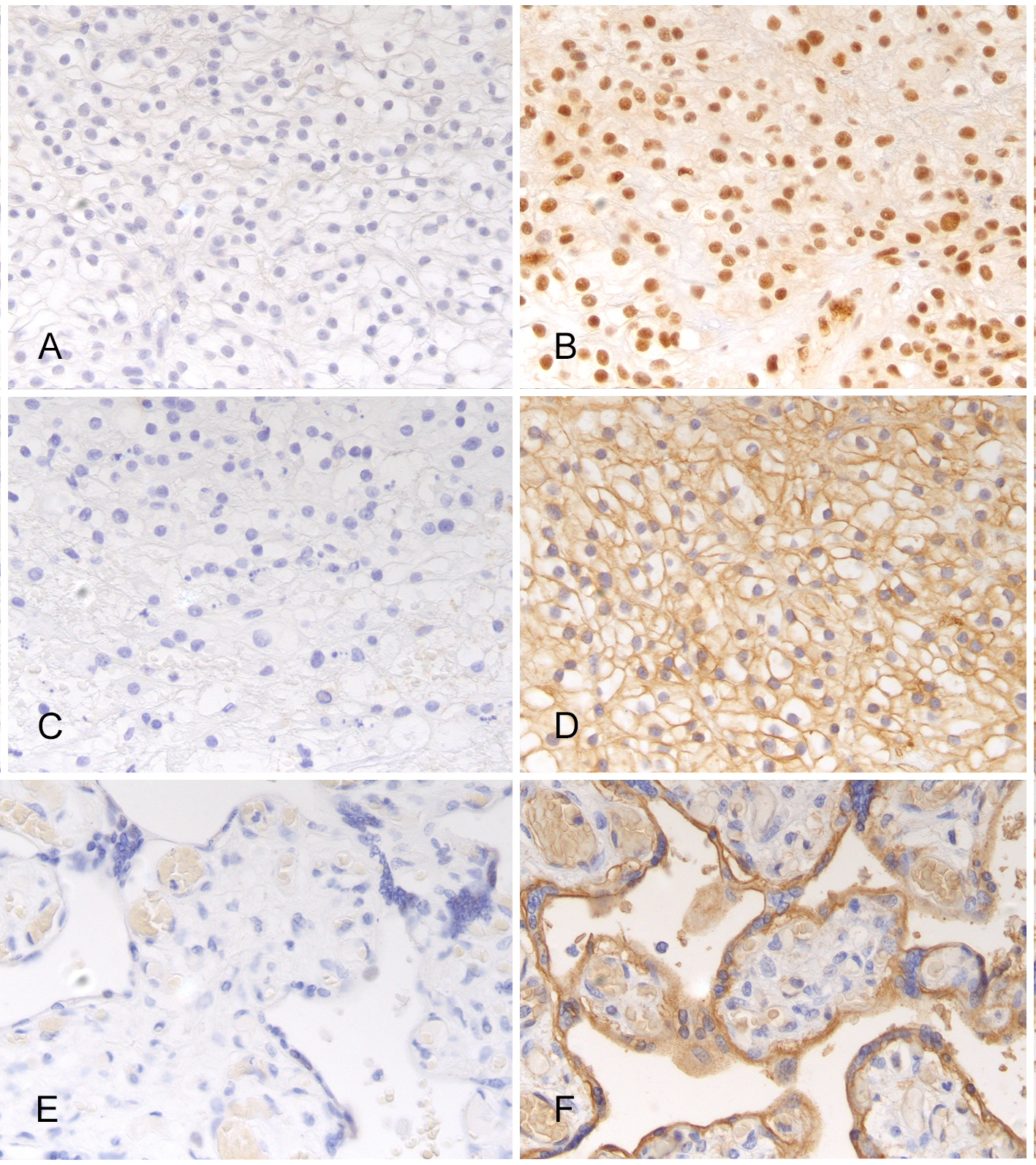

Supplement: Figure S1 — Positive controls: Immunohistochemical staining of HIF-1α and CAIX in renal clear cell carcinoma (B and D) and for Glut-1 in placental tissue (F). In A, C and E the primary antibody was omitted to provide negative controls. (TIF) [file pone.0056055.s001.tif]
